# Nonobese Diabetic (NOD) Mice Lack a Protective B-Cell Response against the “Nonlethal”* Plasmodium yoelii* 17XNL Malaria Protozoan

**DOI:** 10.1155/2016/6132734

**Published:** 2016-12-15

**Authors:** Mirian Mendoza, Luis Pow Sang, Qi Qiu, Sofia Casares, Teodor-D. Brumeanu

**Affiliations:** ^1^Department of Medicine, Division of Immunology, Uniformed Services University of the Health Sciences, Bethesda, MD 20814, USA; ^2^Benaroya Research Institute at Virginia Mason, 1201 Ninth Avenue, Seattle, WA 98101, USA; ^3^Infectious Diseases Directorate, Division of Malaria, Naval Medical Research Center/Walter Reed Army Institute of Research, Silver Spring, MD 20910, USA

## Abstract

*Background*.* Plasmodium yoelii* 17XNL is a nonlethal malaria strain in mice of different genetic backgrounds including the C57BL/6 mice (I-A^b^/I-E^null^) used in this study as a control strain. We have compared the trends of blood stage infection with the nonlethal murine strain of* P. yoelii* 17XNL malaria protozoan in immunocompetent Nonobese Diabetic (NOD) mice prone to type 1 diabetes (T1D) and C57BL/6 mice (control mice) that are not prone to T1D and* self*-cure the* P. yoelii* 17XNL infection. Prediabetic NOD mice could not mount a protective antibody response to the* P. yoelii* 17XNL-infected red blood cells (iRBCs), and they all succumbed shortly after infection. Our data suggest that the lack of anti-*P. yoelii* 17XNL-iRBCs protective antibodies in NOD mice is a result of parasite-induced, Foxp3^+^ T regulatory (Treg) cells able to suppress the parasite-specific antibody secretion.* Conclusions*. The NOD mouse model may help in identifying new mechanisms of B-cell evasion by malaria parasites. It may also serve as a more accurate tool for testing antimalaria therapeutics due to the lack of interference with a preexistent* self*-curing mechanism present in other mouse strains.

## 1. Background

At present, there are no correlates between the trends of malaria infection and autoimmune diabetes, as it is not known whether one disease can worsen the other or, contrarily, may benefit the other disease. Therefore, the main question raised in the present study was whether a predisposing T1D background may interfere with the sensitivity or resistance to malaria infection.

Malaria is an* Anopheles* mosquito-borne infectious disease caused in humans by five different members of the protozoan genus* Plasmodium* (i.e.,* falciparum, vivax, malariae, ovale*, and* knowlesi*).* P. falciparum* is the most virulent and deadly human malaria parasite that annually infects 1 to 2 billion people [1]. In humans, variations in the non-HLA genetic background as well as in the HLA haplotype observed in different ethnic groups were correlated to the sensitivity* versus* resistance to malaria infection [2]. Expression of HLA-DRB1^*∗*^04  alleles has been linked in particular to severe malaria in Gabon and Northern Ghana [3, 4], while the HLA-DRB1^*∗*^1302, HLA-DRB1^*∗*^0101, and HLA-DQB1^*∗*^0501  suballeles have been associated with resistance to severe malaria in The Gambia, Western Kenya, Gabon, and Vietnam [5–8]. In agreement with human studies, we found that, indeed, humanized HLA-DR4 (DRB1^*∗*^0401) mouse lacking murine MHC class II molecules (EA^0^) have impaired production of protective antibodies to nonlethal* P. yoelii* 17XNL strain of malaria, and they succumbed shortly after infection [9].

*Plasmodium yoelii* 17XNL is a nonlethal malaria strain in mice of different genetic backgrounds and MHC class II haplotypes including the C57BL/6 mice (I-A^b^/I-E^null^) used in this study as a control group. Mice show parasitemia shortly upon sporozoites challenge; they gradually develop high titers of antibodies to infected red blood cells (iRBCs) and, as a consequence, they are able to* self*-cure the blood stage infection within 4 to 7 weeks, depending on the sporozoites load and genetic background [9]. In contrast, the NOD mice were unable to mount an antibody response to iRBCs, and they all succumbed shortly after infection.

Several immunodeficient NOD-based models like the NSG model (NOD/Scid mouse) were shown to sustain the* Plasmodium falciparum* blood infection upon infusion with human infected RBCs [10, 11]. However, these models cannot explore a full malaria cycle in vivo, as the liver stage of infection is being bypassed. We have reported that a new humanized HLA-DR4 transgenic NRG mouse was able to sustain a complete vertebrate life cycle of* P. falciparum* malaria [12].

The NOD wild type mouse is a well-known model for spontaneous autoimmune diabetes (Type 1 Diabetes, T1D) in context of several types of immune dysregulation such as impaired macrophage function, reduced Natural Killer (NK) cells and Natural Killer T (NKT) cells, and reduced Treg function [13, 14]. Few weeks after birth, the NOD mice develop prediabetic pancreatic lesions characterized by progressive lymphocyte infiltration of the pancreatic Langerhans (*β*)-islets. Later on, the NOD mice develop high titers of autoantibodies specific for several* self*-proteins including Insulin and GAD65 protein [15]. Hyperglycemia onset occurs in 60–90% of female NOD mice sometimes between 4 and 6 months of age when the pancreatic islets are heavily infiltrated with lymphocytes and more than 80% of the insulin-secreting *β*-cells in the islets are apoptotic. Like in humans, T1D in the NOD mice is an organ-specific autoimmune disease triggered by an unusual high number of* self*-reactive T cells to the pancreatic antigens that bypass the physiological mechanisms of immune tolerance [14].

The NOD wild type mice have been also used to investigate the role of viral infections and trends of autoimmune diabetes [16]. Reports showed that acceleration of T1D in NOD mice and humans is associated with infections with rotaviruses, influenza type A [17–22], and Coxsackie viruses [23–25]. Since T1D not only has a genetic predisposition but is also linked to environmental factors, a number of reports pointed out to environmental infections with bacteria and enteroviruses [18, 26]. While several studies describe the likelihood of EV infections and T1D progression, the results on EVs infection and T1D remain inconclusive and in some instances are even against it [25, 27–30]. Recently, the role of gut microbiota in antigen mimicry of the islet-specific glucose 6-phosphatase catalytic subunit-related protein (IGRP)-reactive CD8 cytolytic cells is believed to play a role in T1D progression in NOD mice [31]. To our knowledge, there are no reports on how a prediabetic or diabetic autoimmune disease can affect the trends of malaria infection in humans or NOD mice.

Herein we investigated the trends of blood stage infection with a “nonlethal” strain of murine malaria (*P. yoelii *17XNL) in immunocompetent, genetically nonmanipulated NOD mice. We report for the first time that the previously denominated “nonlethal” murine strain of* P. yoelii* 17XNL malaria is lethal in NOD mice. Lack of protection and parasite clearance from the blood in the NOD mice was paralleled by the lack of antibody response to* P. yoelii *17XNL-iRBCs. Our data suggest that the CD4^+^Foxp3^+^ T regulatory cells (Treg) may be responsible for a deficient B-cell antibody production specific for* P. yoelii* 17XNL-iRBCs in the NOD mice.

## 2. Methods

### 2.1. Mice

Two-month-old, prediabetic NOD female mice that are prone to the development of autoimmune diabetes and control C57BL/6 female mice that do not develop the disease and are known to* self*-cure the blood stage infection with* P. yoelii* 17XNL parasite were used in the experiments. Mice were purchased from Jackson Labs and housed in a pathogen-free facility at USUHS. The experimental protocol was approved in compliance with Federal and local regulations by the IACUC committee at USUHS.

### 2.2. The Blood Stage Infection with* P. yoelii* 17XNL Sporozoites

Live sporozoites were obtained from the salivary glands of* P. yoelii*-infected* Anopheles stephensi* mosquitoes as we previously described [9]. NOD mice and C57BL/6 mice were challenged retroorbitally with 100* P. yoelii* 17XNL live sporozoites per mouse.* P. yoelii* 17XNL-infected NOD mice and C57BL/6 mice were followed weekly for the trends of blood stage infection based on parasitemia measurements. Parasitemia was monitored 7, 14, 21, 28, and 35 days after challenge by counting 3,000 red blood cells (RBCs) in Giemsa-stained thin blood smears from individual mice and expressed as percentage of infected RBCs (iRBCs), as we previously described [9]. Briefly, Teflon printed slides (12-well; Electron Microscopy Sciences, Hatfield, PA) were coated with iRBCs (10^4^/well) harvested from infected BALB/c, Rag KO mice with parasitemia higher than 30%, and slides were blocked for 30 min at 37°C with phosphate-buffered saline (PBS) containing 1% bovine serum albumin (BSA). Twenty *μ*L of serial dilutions of sera from individual* P. yoelii*-infected mice in each group were added to the wells. Slide smears from these preparations were incubated for 1 h at 37°C, washed three times with PBS, and incubated for 30 min at 37°C with fluorescein isothiocyanate- (FITC-) labeled F(ab′)2 goat anti-mouse total IgG, or IgG1, or IgG2b, or IgG2c, or IgG3 (Southern Biotechnologies, Birmingham, AL).

### 2.3. Diabetes Follow-Up

To find whether the* P. yoelii* 17XNL sporozoites are sequestered in the pancreatic parenchyma or in *β*-islets of the NOD-infected mice and may thus interfere with the early diabetogenic process in pancreas, 5 *μ*m frozen pancreatic sections was incubated for 2 h at 37°C with 1/10 dilution of pooled sera from* self*-cured C57BL/6 mice with high titers of anti-iRBCs IgG2c antibodies. Binding of IgG2c Abs to the pancreatic sections was revealed with an F(ab)′2 goat anti-mouse IgG2c-FITC conjugate, and binding of rabbit anti-insulin Ab was revealed by a goat anti-rabbit IgG-Alexa Fluor 594 conjugate (Life Technologies). Slides were then washed and mounted with Vectashield-DAPI (4′,6-diamidino-2-phenylindole) (Vector Laboratories, Burlingame, CA). Slide preparations were analyzed by fluorescence microscopy, as we previously described [32].

The NOD and C57BL/6 mice challenged with* P. yoelii* 17XNL parasites were monitored weekly for glycemia and development of early pancreatic lesions characteristic of the onset of autoimmune diabetes such as intra- and peri-islet infiltration with lymphocytes. Glycemia was monitored starting 20 days after infection by using an Accu-Check glucose meter and glucose test strips (Roche Co). To identify pancreatic infiltration with lymphocytes and to estimate the amount of intraislet secretion of insulin, 5 *μ*m paraffin-embedded pancreatic sections was doubly stained with hematoxylin-eosin (HE) and with a rabbit anti-insulin Ab revealed by a goat anti-rabbit IgG-HRP (Life Technologies). Some 50 *β*-islets per group of mice were analyzed by fluorescence microscopy for preceding diabetic lesions such as lymphocyte infiltration in the pancreatic *β*-islets.

### 2.4. Analysis of Foxp3^+^ T Regulatory Cells

Single cell suspensions from the spleen of NOD and C57BL/6 mice that were infected or not with* P. yoelii* 17XNL parasites were prepared 20 days after infection. Cells were double-stained with anti-mouse Foxp3 Ab-FITC and anti-mouse CD4-PE conjugates (BD PharMingen, San Jose, CA). Some 2 × 10^5^ cell events were acquired from individual mice in each group and analyzed by a LSR instrument for the frequency of Foxp3^+^ CD4^+^ T cells (Tregs).

### 2.5. Biostatistics

Survival rate of NOD mice and C57Bl/6 mice infected with* P. yoelii* 17XNL parasites was determined by the nonparametric Kaplan-Meier test for which  *p*^*∗*^  values (log-rank Mantel Cox test) of less than 0.05 were considered significant. Significant differences in antibody titers against Py17XNL-iRBCs between the groups of NOD and C57BL/6 mice were measured by the nonparametric test of Mann–Whitney and data were presented as medians with interquartile ranges.

## 3. Results

### 3.1. The NOD Mice Do Not Survive the Blood Stage Infection with the “Nonlethal”* P. yoelii* 17XNL Parasite

**Figure 1:**
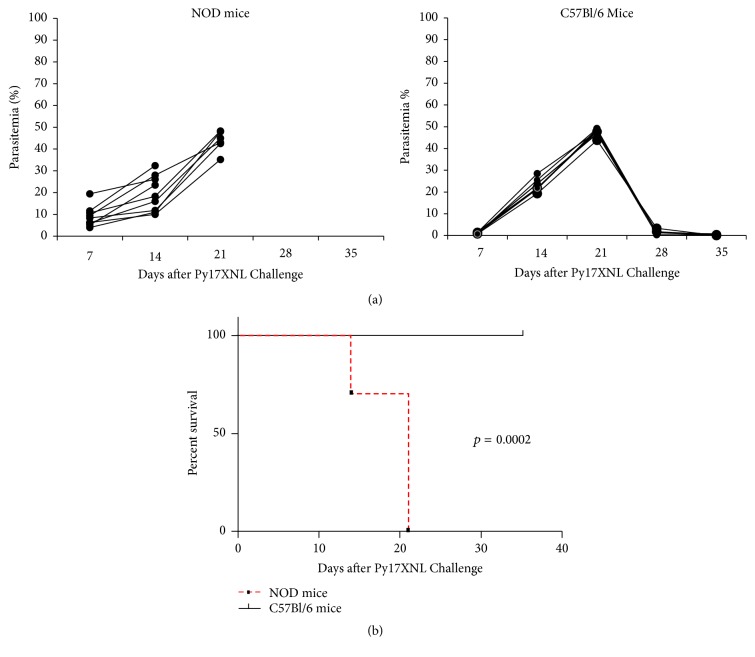
Outcome of* P. yoelii* 17XNL infection in the NOD and C57BL/6 mice. (a) Parasitemia values in 10 NOD mice and 10 C57BL/6 mice after* P. yoelii* infection. The results were collected from two groups of each strain of mice (*n* = 5  mice/group/strain) and represented together on the same graph. Of note, the complete clearance of* P. yoelii* parasites in C57BL/6 mice occurred by day 35 after infection. The degree of variability between the analyzed groups was null, since all NOD mice succumbed to the infection and all C57BL/6 mice* self*-cured the infection regardless of the time when the experiments were carried out. (b) The percentage of survival in NOD and C57BL/6 groups challenged with* P. yoelii* 17XNL sporozoites.

*P. yoelii* 17XNL strain of malaria is nonlethal in various mouse strains including the C57BL/6 mouse strain. Mice infected with* P. yoelii* 17XNL go through a transitory stage of infection, as they totally clear the parasites and* self*-cure within 4 to 6 weeks, depending the parasite load and genetic background. The NOD wild type mouse is a mouse model for spontaneous autoimmune diabetes (T1D) in which the outcome of infection with the nonlethal* P. yoelii *17XNL strain after infection has not been investigated yet. Herein, we tested for the first time whether the NOD mouse* self*-cures the infection with nonlethal* P. yoelii *17XNL malaria parasite. Two large groups of 2-month-old NOD female mice (MHC class II haplotype  I-A^g7^/I-E^null^) and C57BL/6 female mice (MHC class II haplotype  I-A^b^/I-E^null^) were challenged with 100 infectious* P. yoelii* 17XNL sporozoites per mouse. Both groups of mice started to show blood stage parasitemia within 14 days after challenge (Figures 1(a) and 1(b)). The reproducibility of results within the same group of mice was statistically significant, and the degree of variability among individual mice within the same group was insignificant according to the nonparametric Kaplan-Meier test.

C57BL/6 mice have been chosen as a control group that* self*-cure the blood stage infection with nonlethal strain of* P. yoelii* 17XNL protozoan. The rate of survival in C57BL/6 group was significantly higher than in the NOD group 14 days after challenge (^*∗*^*p* = 0.0002, Figure 1(b)). In contrast to the C57BL/6 mice that* self*-cured the blood stage infection with* P. yoelii *17XNL by day 35 after infection, the NOD mice did not. The NOD-infected mice started to succumb by day 14 after challenge when parasitemia reached 10% to 30% (Figure 1(a)). No NOD-infected mouse survived longer than 21 days after challenge when parasitemia was close to 50%. In contrast, all C57BL/6 mice with similar levels of parasitemia 21 days after challenge survived, and they were able to completely clear the parasites by day 35 after challenge (Figure 1(b)).

### 3.2. *P. yoelii* 17XNL-Infected NOD Mice Cannot Mount an Anti-iRBCs Antibody Response

**Figure 2:**
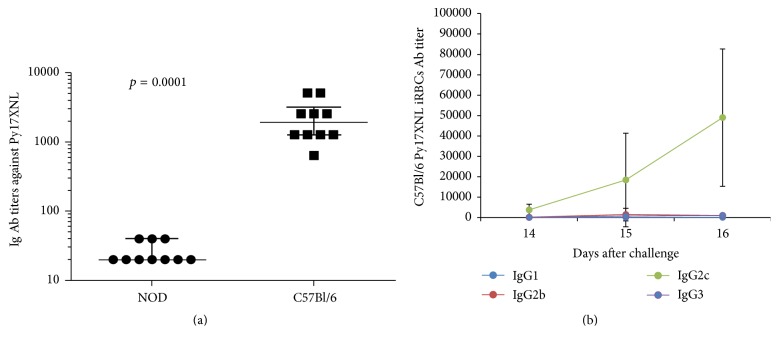
Anti-iRBCs B-cell responses in the* P. yoelii* 17XNL-infected NOD and C57BL/6 mice. (a) Antibody titers in individual mice from* P. yoelii-*infected NOD mice and C57BL mice by immunofluorescence (IFA) 14 days after infection; (b) sera from the same individual mice analyzed at the same time points as in (a) for the IgG1, or IgG2b, or IgG2c, or IgG3 titers specific for iRBCs. *y*-axis shown in both (a) and (b) represents the serum serial dilutions tested in IFA. Of note, only specific IgG2c antibodies were detected in the blood of C57BL/6 mice, but not in NOD-infected mice. The results were collected from each strain of mice (*n* = 5 mice/group/strain).

In most mouse strains including the C57BL/6 mice (control mice in these experiments) the specific antibodies are critical for clearing the* P. yoelii* 17XNL malaria parasites from the blood [9, 33]. We previously showed that the process of* self*-curing the blood stage infection with* P. yoelii* 17XNL parasite by C57BL/6 mice is strongly dependent on the IgG2c antibody titer to the* P. yoelii*-iRBCs [9]. In contrast to the C57BL/6 mice, none of the NOD mice was able to mount a specific antibody response against the* P. yoelii* 17XNL-iRBCs, and they all succumbed to the parasite infection. Thus, 14 days after challenge the titer of antibodies specific for iRBCs was significantly higher in* P. yoelii* 17XNL-infected C57BL/6 mice than in* P. yoelii* 17XNL-infected NOD mice according to the Mann–Whitney test (^*∗*^*p* = 0.0001) (Figure 2(a)). The predominant class of antibodies specific for* P. yoelii* 17XNL-iRBCs in C57BL/6 mice was IgG2c (Figure 2(b)).

### 3.3. *P. yoelii* 17XNL-Infected NOD Mice Upregulate the Size of Foxp3^+^ CD4^+^ T Regulatory Cell Pool

**Figure 3:**
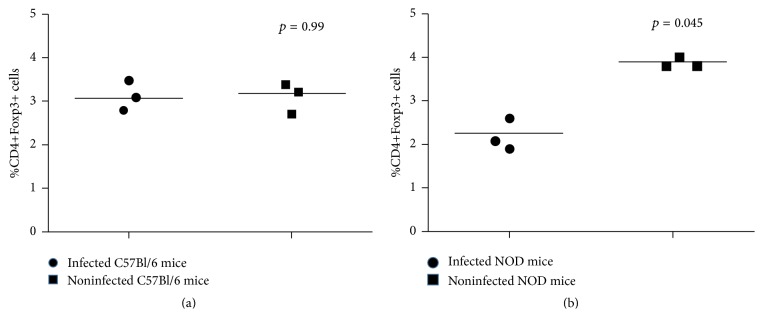
Frequency of Foxp3^+^ CD4^+^ T regulatory cells (Tregs) in* P. yoelii* 17XNL-infected NOD mice. Cytofluorimetric analysis of Tregs was determined by FACS in the spleen cells from individual mice from both groups (*n* = 3  mice/group) that were not infected or infected with* P. yoelii* 17XNL live sporozoites. FACS measurements were carried out 20 days after infection. (a) Percent of peripheral (splenic) Tregs in 3 individual mice from C57Bl/6 control mice (*n* = 3) before and after each* P. yoelii* 17XNL challenge; group; (b) percent of peripheral (splenic) Tregs in the spleen of NOD mice (*n* = 3) before and after* P. yoelii* 17XNL challenge. Shown are the significance  ^*∗*^*p*  values between the groups of infected and noninfected mice.

FACS measurement of Foxp3^+^ Tregs frequency in the blood of infected NOD mice at day 14 after infection showed a 50% to 65% increase as compared to their own controls prior to infection. Some 20 days after infection the size of Foxp3^+^ Tregs pool in* P. yoelii* 17XNL-infected NOD mice was significantly increased by almost 100% (^*∗*^*p* = 0.045, Figure 3(b)). In agreement with our previous study [9], the* P. yoelii* 17XNL-infected C57BL/6 mice showed no significant increase in the size of Foxp3^+^ Tregs pool (^*∗*^*p* = 0.99) (Figure 3(a)). Accordingly, a significant increase in the number of Foxp3^+^ Tregs strongly suggests that these cells may well account for the suppression of antibody response to* P. yoelii* 17XNL-iRBCs, which in turn explains the lack of protection in NOD mice.

### 3.4. *P. yoelii* 17XNL Infection Does Not Accelerate the Prediabetogenic Process in the NOD Mice

**Figure 4:**
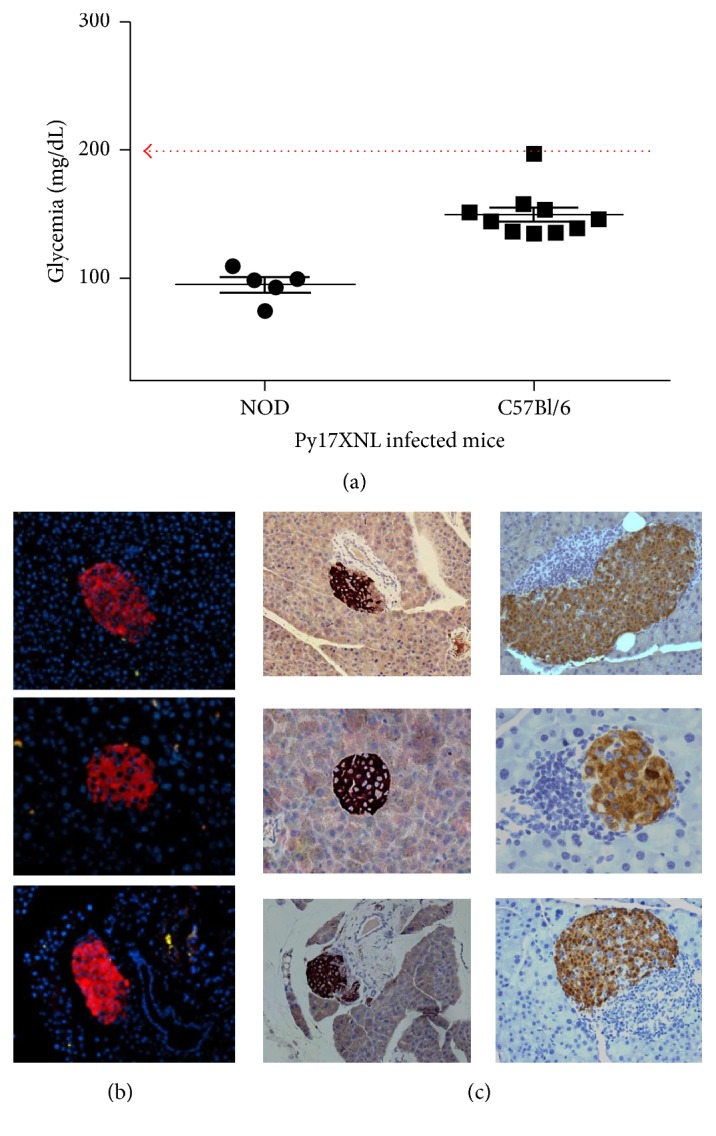
Pancreatic analysis of NOD mice infected with* P. yoelii* 17XNL sporozoites. (a) Glycemia values in* P. yoelii* 17XNL-infected mice as monitored 20 days after infection. Dotted line indicates the upper limit of normoglycemia (200 mg/dL) as determined in a cohort of 25 untouched, 2-month-old C57Bl/6 mice and 20 untouched, 2-month-old NOD mice; (b) pancreatic *β*-islets from* P. yoelii*-infected NOD mice at day 20 after infection. Shown are scattered* P. yoelii* 17XNL-iRBCs (green spots) in the pancreatic parenchyma. The *β*-islets are shown in fluorescent red and cell nuclei in blue. (c) Three representative pancreatic *β*-islets from a* P. yoelii*-infected NOD mouse that succumbed 21 days after infection (left panels). Note the lack of lymphocyte infiltration and normal intraislet insulin secretion (dark-brown color) in contrast to heavy lymphocyte infiltration (dark cyan cells) and reduced intraislet insulin secretion in a 5-month-old, diabetic NOD control mouse (3 right panels).

Infection of NOD mice with* P. yoelii* 17XNL did not accelerate the T1D development in the prediabetic stage, as the* P. yoelii* 17XNL infection did not show an earlier onset of pancreatic infiltration with lymphocytes (pancreatic insulitis, Figure 4(a)). With the exception of few* P. yoelii* 17XNL-infected RBCs (iRBCs) found in the pancreatic vessels, there was no sequestration of iRBCs in the pancreatic parenchyma or *β*-islets (Figure 4(b)). Pancreatic *β*-islet infiltration with lymphocytes in humans as well as in NOD mice is an early prediabetic lesion mediated by autoreactive T cells that precedes the onset of hyperglycemia. Also, pancreatic *β*-islet infiltration with lymphocytes in the NOD mice with high parasitemia (30–50% parasitemia) at day 20 after infection has not been detected (Figure 4(c), left panel). These results indicated that the* P. yoelii* 17XNL-infected NOD mice did not show an accelerated onset of pancreatic insulitis.

## 4. Discussion

In our knowledge, there are no reports describing the outcomes of malaria infection in mice or in humans predisposed to autoimmune diabetes. Herein, we have investigated prediabetic NOD mice as the most appropriate model for human autoimmune diabetes (type 1 diabetes, T1D) [15]. Our data showed for the first time that NOD mice prone to autoimmune diabetes cannot* self-*cure the blood stage infection with a “nonlethal” strain of murine malaria (*P. yoelii* 17XNL), as they cannot mount a protective antibody response to the parasite. The results of this study suggest that several immune evasion mechanisms may operate alone or together in* P. yoelii* 17XNL-infected NOD mice.

Different murine genetic backgrounds respond to different strains of malaria infection with different antiparasite antibody classes, which depends in part on the degree of T cell involvement. Thus, the IgG1 isotype is sustained by CD4 Th2 cells, while the IgG2a is sustained by CD4 Th1 cells. As we previously reported [9], the C57BL/6 mouse responds to P.* yoelii* infection with an IgG2a-like isotype (IgG2c) [34] that is a Th1-biased response. Since the NOD mice share much of the C57BL/6 genetic background, they can also secrete an IgG2a-like isotype (IgG2c). The IgG2c antibodies to* P. yoelii* 17XNL-iRBCs are known to play critical role in clearing the parasite and* self*-curing the infection by mice with different genetic backgrounds [9]. The inability of NOD mice to surpass the blood stage infection and* self*-cure the* P. yoelii* 17XNL infection was strongly associated with the lack of a specific antibody response to the parasite-iRBCs. Lack of a specific antibody response in the NOD mice is not a consequence of deficient B-cell function, since during the diabetes development these mice develop high titers of autoantibodies to a large number of* self*-antigens including Glutamic Acid Decarboxylase of 65 KDa (GAD65) and Insulin [15]. In addition, we have reported that immunization of prediabetic NOD mice with a preparation of UV-inactivated influenza virus PR8/A/34 induced a robust titer of neutralizing anti-viral antibodies [35]. Recently, it has been shown that the influenza virus infection does not impair the antiviral immune response in NOD mice [36]. Also, human studies showed no significant difference in the titers of anti-influenza viral antibodies between nonvaccinated, genetically predisposed children to T1D and control children [37]. Our study showed that in the case of* P. yoelii* 17XNL infection, the NOD mice are unable to mount an anti-parasite antibody response, which infers our previous hypothesis that a major mechanism of immune evasion by malaria parasites relies on suppression of B-cell function throughout induction of parasite-specific Foxp3^+^ Treg cells [38].

We recently reported that specific B-cell responses to the* P. yoelii* 17XNL-iRBCs are efficiently suppressed by Foxp3^+^ Treg cells in a C57BL/6 mouse coexpressing a human MHC class II molecule (HLA-DR^*∗*^0401  transgene), but not in C57BL/6 mice coexpressing other HLA transgenes [9]. Obviously, induction of Tregs by the* P. yoelii* 17XNL parasite is tightly controlled by some MHC class II haplotypes. The inability of NOD mice to develop anti-*P. yoelii *17XNL-iRBCs antibodies occurred in the context of a significant increase (almost 100%) in the number of splenic Foxp3^+^ Treg cells. Quite interestingly, the Treg compartment known to control the T cell homeostasis including the diabetogenic CD4 T cells, is deficient in the NOD mice [13, 39] as compared with mouse strains not prone to autoimmune diabetes. This strongly suggests that the increase size in the pool of Foxp3^+^ Tregs in* P. yoelii* 17XNL-infected NOD mice was mainly induced by the parasite. It thus remains to define what parasite antigens at what stage of infection are responsible for upregulation of parasite-specific Foxp3^+^ Tregs. This is a difficult task to achieve if taking into account the large number of antigens expressed by the parasite during its life cycle. Theoretically, adoptive cell transfer of Tregs from* P. yoelii* 17XNL-infected NOD mice to C57BL/6 mice prior to infection with* P. yoelii* 17XNL is expected to lower the resistance of C57BL/6 mice to the infection, and it would provide direct evidence of Tregs suppression of anti-*P. yoelii* protective antibodies. However, technically, an adoptive cell transfer is not be feasible due to the development of host versus graft reaction. Using an alternative approach, we showed that depletion of Foxp3^+^ Treg cells with an anti-CD25 antibody in HLA-DR transgenic C57BL/6 mice (unable to raise IgG2c anti-*P. yoelii*-iRBCs antibodies and could not* self*-cure the infection) enables these mice to raise anti-parasite antibodies and to* self*-cure the blood stage infection [9].

The MHC class II molecules on antigen presenting cells bind peptide antigens and present them to various T cell subsets, a process leading to cell activation and expansion depending on the structure of peptide and MHC class II molecules. The MHC class II haplotype in NOD mice and C57BL/6 control mice used in this study is quite different: I-A^g7^/I-E^null^* versus *I-A^b^/I-E^null^. Likewise, the MHC class II I-A^g7^ molecules in NOD mice and MHC class II HLA-DQ8 molecules in humans have the same single-point mutation at Asparagine 56 in the variable region of I-A *β*-chain, and both molecules are associated with high predisposition to T1D [40]. The Asp56 amino acid residue has been considered to provide unusual binding and presentation of* self*-peptides like some pancreatic* self*-peptides to the CD4 T helper cells, which in turn may trigger expansion of pancreatic* self*-reactive (diabetogenic) CD4 Th1-cells and ultimately the development of autoimmune diabetes [40]. It remains to further investigate if the unique structure of the I-A^g7^ molecules expressed by the NOD mice may present* P. yoelii* 17XNL-derived peptides able to expand the population of* P. yoelii* 17XNL-specific Foxp3^+^ Tregs that can lead to suppression of the antiparasite specific B-cell response. The MHC class II (I-A^g7^) haplotype in the NOD mouse is unique, and there is no other mouse MHC class II haplotype that develops spontaneous diabetes. However, the I-A^g7^ haplotype NOD mouse cannot be completely ruled in as the main cause for lack of resistance to P. yoelii infection as long as the gene polymorphism in more than 22 genes associated with diabetes is ruled out.

## 5. Conclusions

Prediabetic* P. yoelii* 17XNL-infected NOD mice were unable to mount an anti-parasite antibody response and could not* self*-cure the blood stage infection. Accordingly, one may reconsider the terminology of “nonlethal” for the* P. yoelii* 17XNL strain of murine malaria. Our data suggest that the immune evasion by* P. yoelii 17XNL *parasite in the NOD mice may well be the parasite-induced Foxp3^+^ CD4^+^ Treg cells able to suppress the parasite-specific B-cell responses. On the autoimmune side of this study, the* P. yoelii* 17XNL infection did not seem to accelerate the early diabetogenic process in pancreas. The NOD mouse model may help in identifying new mechanisms of B-cell evasion by malaria parasites. The NOD mouse may also serve as a more accurate tool for testing antimalaria therapeutics due to the lack of interference with a preexistent* self*-curing mechanism present in other mouse strains.

## Abbreviations

DAPI: 4′,6-Diaminodino-2-phenylindole
FITC: Fluorescein isothiocyanate
HE: Hematoxylin-eosin stain
HLA: Human leukocyte antigen
iRBCs: Infected red blood cells
MHC: Major histocompatibility complex
NOD mice: Nonobese Diabetic mice
Tregs: T regulatory cells
T1D: Type 1 diabetes, autoimmune diabetes.
